# Sarcomere Lattice Geometry Influences Cooperative Myosin Binding in Muscle

**DOI:** 10.1371/journal.pcbi.0030115

**Published:** 2007-07-13

**Authors:** Bertrand C. W Tanner, Thomas L Daniel, Michael Regnier

**Affiliations:** 1 Department of Bioengineering, University of Washington, Seattle, Washington, United States of America; 2 Department of Biology, University of Washington, Seattle, Washington, United States of America; University of Washington, United States of America

## Abstract

In muscle, force emerges from myosin binding with actin (forming a cross-bridge). This actomyosin binding depends upon myofilament geometry, kinetics of thin-filament Ca^2+^ activation, and kinetics of cross-bridge cycling. Binding occurs within a compliant network of protein filaments where there is mechanical coupling between myosins along the thick-filament backbone and between actin monomers along the thin filament. Such mechanical coupling precludes using ordinary differential equation models when examining the effects of lattice geometry, kinetics, or compliance on force production. This study uses two stochastically driven, spatially explicit models to predict levels of cross-bridge binding, force, thin-filament Ca^2+^ activation, and ATP utilization. One model incorporates the 2-to-1 ratio of thin to thick filaments of vertebrate striated muscle (multi-filament model), while the other comprises only one thick and one thin filament (two-filament model). Simulations comparing these models show that the multi-filament predictions of force, fractional cross-bridge binding, and cross-bridge turnover are more consistent with published experimental values. Furthermore, the values predicted by the multi-filament model are greater than those values predicted by the two-filament model. These increases are larger than the relative increase of potential inter-filament interactions in the multi-filament model versus the two-filament model. This amplification of coordinated cross-bridge binding and cycling indicates a mechanism of cooperativity that depends on sarcomere lattice geometry, specifically the ratio and arrangement of myofilaments.

## Author Summary

Striated muscle is highly structured, and the molecular organization of muscle filaments varies within individuals (by fiber type) and taxonomically. The consequences of filament arrangement on muscle contraction, however, remain largely unknown. We explore how filament arrangement affects force production in muscle using spatially explicit models of many interacting myofilaments. Our analysis incorporates molecular scale force balance equations with Monte Carlo simulations of both actin–myosin interactions and thin-filament Ca^2+^ activation. Simulations show that a more physiological representation of vertebrate striated muscle amplifies force production, coordinates dynamic actin–myosin cycling, and may optimize energetics of contraction (force generated per ATP consumed). This coordinated myosin behavior indicates a mechanism of cooperativity in muscle that depends on the ratio and arrangement of filaments. We also demonstrate the importance of mechanical coupling between myosin molecules by varying filament stiffness. Our simulations show a tradeoff between the way myosin molecules partition energy from ATP hydrolysis into force transmitted throughout the filaments versus distortions within the filaments. These findings present a possible consequence of organization in muscle, where the ratio and arrangement of muscle filaments affects contractile performance for the given function across different muscle types.

## Introduction

Muscle contraction is initiated by Ca^2+^ binding to troponin and the subsequent movement of tropomyosin on the thin filament, enabling myosin to cyclically attach and detach to actin (cross-bridge cycling) [1–7]. Underlying this process are myriad factors that contribute to the magnitude and time course of force production. These factors include the geometry of filaments in the sarcomere, the mechanical properties of the filaments and cross-bridges, the kinetics of thin-filament activation by Ca^2+^, and the kinetics of cross-bridge cycling. Because contractile proteins interact in a highly structured, compliant lattice, mechanical coupling exists between myosins along the thick-filament backbone, between actin monomers or regulatory proteins (troponin and tropomyosin) along the thin filament, and between thick and thin filaments following cross-bridge formation. Thus, kinetic processes responsible for contraction are linked at the molecular level. Considerable evidence shows that Ca^2+^ and cross-bridge binding at one location in the sarcomere can influence these processes at proximal regions of the sarcomere (reviewed in [7]), implying that coupled kinetics of thin-filament activation and cross-bridge cycling determine the level of force generated in striated muscle.

Most models do not explicitly consider that spatial properties of muscle may influence contraction [1,3,4,6,8–12]. Of the muscle contraction models containing both spatial and temporal variables, some provide either spatial predictions of steady-state conditions [9,13] or temporal predictions of cross-bridge and thin-filament “state” without any spatial detail [12]. In contrast, a few recent spatially explicit models predict both spatial and temporal behavior [14–18], with some simulations indicating that elasticity of the myofilament lattice contributes to coordination between cross-bridges that enhances cross-bridge binding [15,17,18]. This cross-bridge–induced cross-bridge recruitment becomes a potential mechanism of cooperativity that results from realignment between compliant myofilaments following myosin binding to actin. Previous spatially explicit models [14–17] lacked a Ca^2+^ regulatory cycle, spatially coordinated Ca^2+^ activation along the thin filament, and the physiological ratio of thick to thin filaments. These thin-filament components are particularly important for contraction because regions activated by Ca^2+^ binding to troponin largely determine the spatial distribution of bound cross-bridges. The current study adopts a spatially explicit model of regulatory proteins along the thin filament, in contrast to prior studies [17,18]. These additions enable investigating how force is controlled by two, coupled, spatial, and temporal processes: Ca^2+^ binding to activate the thin filament and subsequent myosin binding to the proximal, activated region of the thin filament.

Spatial and temporal aspects of contraction may be profoundly influenced by the coupled behavior between myosins throughout the compliant myofilament lattice, as nearly 70% of muscle compliance resides in the thick and thin filaments [19–23]. This significant compliance implies that cross-bridges do not operate independently while generating force [15,17,18]. Moreover, recent measurements [24–26] improve estimates about cross-bridge rate functions, depending on distortion and load, suggesting that the extent of realignment between compliant thick and thin filaments may affect kinetics of cross-bridge cycling (in addition to number of bound cross-bridges). Within this compliant system, however, the consequences of sarcomere lattice structure on cross-bridge dynamics remain unclear.

**Figure 1:**
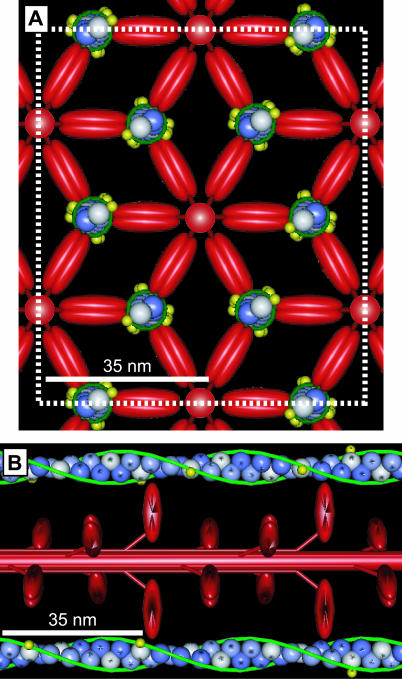
Multi-Filament Geometry The multi-filament model comprises four thick and eight thin filaments. (A) A cross-sectional representation of model geometry shows thick filaments in red and thin filaments in blue. Toroidal boundary conditions (outlined by the dotted white square) reflect the behavior at each edge onto the opposite edge of the simulation space. This condition permits simulating a subsection of infinite lattice space without any edge effects. (B) A truncated side view represents a single thick filament with two co-linear facing thin filaments in the plane of the page. Myosin extends from the central body of the thick filament. Thin filaments show each actin strand in a different shade of blue, with white actin monomers representing the actin nodes in the model. The proteins troponin (yellow) and tropomyosin (green) are located along each actin strand to provide Ca^2+^-sensitive regulation of actin and myosin binding.

**Figure 2:**
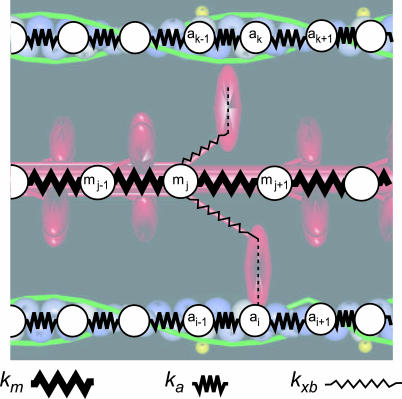
Filament Mechanical Interactions Mechanics are simulated using a network of linear springs, with tunable spring constants *k_m_*, *k_a_*, and *k_xb_* for the thick filament, thin filament, and myosin cross-bridge. *m_j_* through *m_j_*_+1_ are thick-filament nodes, with actin nodes *a_i_*_−1_ through *a_i_*_+1_ and *a_k_* through *a_k_*_+1_ along opposing thin filaments. We show only those cross-bridge spring elements extending from *m_j_* to illustrate a co-linear plane of filament interactions; all other cross-bridges lie outside of this plane. Thus, binding between *m_j_* and *a_i_* occurs when: 1) *a_i_* is activated by Ca^2+^ to bind with myosin, 2) the cross-bridge is in the proper rotational plane, and 3) both nodes are close enough to permit a reasonable probability of cross-bridge binding.

**Figure 3:**
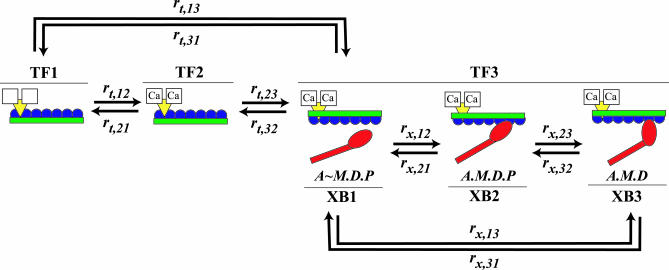
Kinetic Scheme Model kinetic structure (using the same color scheme as Figure 1) shows coupled, three-state cycles for thin-filament activation and cross-bridge formation. Thin-filament states TF1, TF2, and TF3, represent no Ca^2+^ bound to troponin, Ca^2+^ bound to troponin, and Ca^2+^ bound to troponin plus a movement of tropomyosin that exposes myosin binding sites on actin, respectively. TF1 and TF2 represent thin-filament conformations where myosin cannot bind to actin. Cross-bridge states XB1, XB2, and XB3 represent unbound, bound pre-powerstroke, and bound post-powerstroke actomyosin conformations, respectively. Cross-bridge conformations associated with XB2 and XB3 bear force. We list a possible biochemical condition associated with each cross-bridge state using *A*, *M*, *D*, and *P* to represent actin, myosin, ADP, and inorganic phosphate (*P_i_*). *A ∼ M* signifies the unbound state (XB1), and *A* . *M* represents actomyosin binding (XB2 and XB3). While transition rates between cross-bridge states (*r_x,ij_*) depend on position and cross-bridge distortion, the transition rates between thin-filament states (*r_t,ij_*) do not. Note that the transition between XB3 and XB1 is biochemically associated with a release of ADP, myosin binding another ATP, dissociation of myosin from actin, and hydrolysis of ATP into products ADP and P*_i_*. Thin-filament transitions from TF3 to TF2 are not permitted while a cross-bridge is bound. Each cycle is thermodynamically balanced (Equations 7 and 14).

This study compares multiple models that have identical thin-filament and cross-bridge kinetics, but different model geometries, to examine the consequences of sarcomere lattice structure on Ca^2+^-regulated contraction (Figure 1, see Methods section). Consistent with previous models [15,17,18], motions and forces occur solely along the longitudinal axis of filaments in these current models (Figure 2). This one-dimensional assumption permits a system of linear equations to describe force-generating interactions between filaments (Equations 2–4). At the core of each model is a three-state cross-bridge cycle coupled with a three-state thin-filament regulatory model to control actomyosin binding through [Ca^2+^]-sensitive kinetics (Figure 3). Initial model comparisons occurred between four different models (see Text S1). Of these geometric options, only one multi-filament model yields predictions that were consistent with the range of published values for muscle contraction (see Table S1). Therefore, we focus on comparing this multi-filament model (Figure 3) with a two-filament model (*sensu* [15]).

Throughout this study, we specifically consider contraction in the absence of cooperative, kinetic feedback between thin-filament activation or cross-bridge binding [11,12,15,27–29]. Thus, any differences in simulation predictions between the multi-filament and two-filament models depend solely on differences between model geometry. Simulation results show that additional inter-filament interactions in the multi-filament model lead to greater fractional binding of cross-bridges, force production, and cross-bridge turnover compared with the two-filament model. Importantly, these increases are larger than predicted by normalizing for the additional filaments in the multi-filament model. These results indicate that there is a mechanism of cooperativity dependent upon sarcomere lattice structure (both the ratio and arrangement of myofilaments). Specifically, multi-filament lattice structure further coordinates cross-bridge binding to enhance cross-bridge recruitment and turnover without any requirements for cooperative feedback mechanisms attributed to thin-filament activation. Additional studies investigating other mechanisms of cooperativity acting via kinetic feedback pathways to amplify thin-filament activation or cross-bridge binding are ongoing in our lab and in others [30,31]. Findings from the current study, however, imply that certain lattice geometries facilitate greater cross-bridge binding and turnover, which may be an important mechanism of cooperativity contributing to muscle performance. Earlier aspects of this work have been published previously [30,32,33].

## Results

Three principle conclusions follow from our analysis of the multi-filament and two-filament models: 1) the multi-filament model simulates literature values of skeletal muscle force, ATPase, and cross-bridge binding better than the two-filament model; 2) in the absence of a cross-bridge feedback on thin-filament activation, there is no difference in Ca^2+^ sensitivity between the two models; and 3) multi-filament model geometry amplifies the influence of filament compliance on cross-bridge binding and turnover.

### Dynamic Behavior

**Figure 4:**
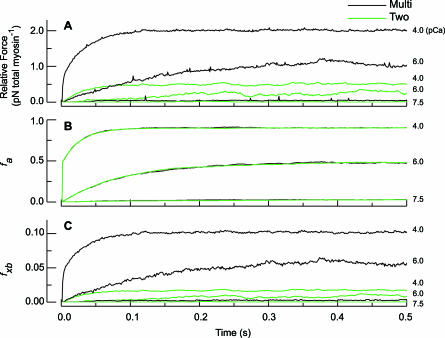
Transient Predictions Temporal predictions of average relative force (A), fractional thin-filament nodes available to bind with myosin, *f_a_* (B), and fractional cross-bridge binding, *f_xb_* (C) for the multi-filament (black) and two-filament (green) models at pCa levels 4.0, 6.0, and 7.5 (pCa = −log_10_ [Ca^2+^]). The number of simulations trials averaged to generate each trace (*N_runs_* or 24*N_runs_*) is summarized in Table 3. Our standard mechanical parameters apply to these simulations: *k_xb_* = 5, *k_a_* = 5229, and *k_m_* = 6060 pN nm^−1^.

These models provide both temporal and spatial predictions for force, cross-bridge binding, thin-filament Ca^2+^ activation (fraction-available actin nodes), and ATP consumption via cross-bridge turnover. Temporal dynamics of these predictions highlight similarities and differences between the simpler two-filament model and the multi-filament model (Figure 4). Although the multi-filament and two-filament models show similar thin-filament activation dynamics that lead to greater magnitude and rate of force generation with increasing [Ca^2+^], the multi-filament geometry produces higher force and signal-to-noise ratio. Maximal, average, steady-state force (at pCa 4, where pCa = −log_10_[Ca^2+^]) for the multi-filament model is 958.5 ± 32.3 pN, compared with 9.2 ± 8.2 pN for the two-filament model (mean ±SD). This ≈100-fold increase in force occurs even though the potential inter-filament interactions in the multi-filament model increase only 24 times.

Relative force (ratio of predicted force to total myosin) adjusts for the relative number of potential interactions between models and is about 4-fold greater (=100/24) in the multi-filament model (Figure 4A). The maximal relative force value predicted by the multi-filament model (≈2 pN myosin^−1^ at pCa 4, Figure 4A) lies in the range (≈1–3 pN myosin^−1^) estimated from experimental studies [25,34,35]. Isometric force measurements from single fibers set the low end of this value at 1–1.7 pN myosin^−1^ [34,35], depending upon temperature and estimated myosin binding (*f_xb_*). Rescaling the value of 1.4 pN per head [25] from single-molecule studies sets the upper end of this range at 2.8 pN myosin^−1^. The predicted relative force values by the two-filament model (Figure 4A) are below this range. Furthermore, tensile stress (force per cross-sectional area) for the multi-filament model, 171 ± 6 kPa, is also consistent with literature values (Table S1, see Methods section for calculated area = 5,600 nm^2^). Collectively, these results suggest that the more physiological lattice geometry in the multi-filament model introduces a geometry-dependent increase in predicted force values, agreeing better with experimental force values than the two-filament model.

Comparing relative force traces across multiple [Ca^2+^] (Figure 4A) shows more variation in the two-filament model, even though these traces average 24 times as many simulation runs as multi-filament traces (see Methods section). Although the multi-filament traces show less relative variation in force level, these traces have occasional “spikes” not present in the two-filament traces due to increased two-filament averaging. Greater variation in the two-filament model results from a lower number of cross-bridge interactions between the filaments. Additionally, force-generating events are less frequent in the two-filament model, giving each of these events more influence on the force level.

Thin-filament activation does not depend on geometry differences between the two models (largely because this study does not examine cross-bridge feedback increasing thin-filament activation). Thin-filament activation contrasts with force production and cross-bridge binding, which both depend upon the coupled probability of myosin binding a proximal actin node with the probability of that actin node being activated by Ca^2+^. The fraction of thin-filament sites available to bind myosin (*f_a_*) is calculated from the number of actin nodes populating state TF3 divided by the total number of actin nodes. Magnitude and rate of *f_a_* increase with [Ca^2+^] (Figure 4B), and maximal steady-state *f_a_* (pCa 4) for the multi-filament and two-filament models is 0.90 ± 0.01 and 0.90 ± 0.03, respectively. Given identical thin-filament activation kinetics in both models, with no influence of cooperative feedback between the kinetics of thin-filament activation and cross-bridge cycling in either model, we expect similar thin-filament activation dynamics. Stochastic variation in *f_a_* is slightly less in the multi-filament versus the two-filament model, likely because the increased number of actin nodes decreases the influence of any single actin node. For each model, variation in *f_a_* is smaller than variation in the corresponding force trace at a similar [Ca^2+^]. This decreased variance in *f_a_* (compared with force) occurs because Ca^2+^-binding kinetics assume a spatially homogeneous [Ca^2+^] within the cell (in contrast to the spatial constraints of cross-bridge binding and force generation). Although these differences leading to thin-filament activation and force production may appear subtle, they demonstrate that spatially explicit implementation of thin-filament activation is critical for investigating the molecular mechanisms controlling force production.

Lower fractional myosin binding (*f_xb_*) in the two-filament model is not limited by *f_a_*, which implies differences in cross-bridge binding stem purely from geometry differences between the two models (Figure 4C). *f_xb_* is calculated from the sum of cross-bridges in states XB2 and XB3 divided by the total number of cross-bridges. Similar to force and *f_a_*, the magnitude and rate of *f_xb_* increases with increasing [Ca^2+^]. Maximum *f_xb_* (pCa 4) is 0.101 ± 0.002 and 0.015 ± 0.014 for the multi-filament and two-filament models, respectively. This predicted multi-filament value is near the low end of estimates [34–36], and stiffness properties of the lattice or kinetic feedback may augment this value (discussed below in detail).

### Steady-State Behavior

**Figure 5:**
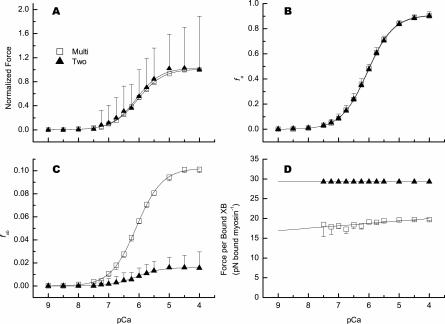
Steady-State Predictions Average force (A), fractional thin-filament activation, *f_a_* (B), fractional cross-bridge binding, *f_xb_* (C), and calculated force per bound myosin cross-bridge (D) from the multi-filament (open square) and two-filament (black triangle) models are plotted over the range of simulated [Ca^2+^]. Predictions are reported as mean ±SD, where single-sided error bars project downward for the multi-filament versus upward for the two-filament model. Error bars lie within the symbol when not visible. Mechanical parameters for *k_xb_*, *k_a_*, and *k_m_* are the same as those outlined in the legend of Figure 4. In Figure 4A−4C, the solid lines represent least squares minimization of the data to a three-parameter Hill equation (Equation 22). Force-per-bound cross-bridge is not calculated for low [Ca^2+^] levels where extremely limited cross-bridge binding occurs. The solid lines in (D) are least squares fits to the data.

Steady-state predictions (mean ±SD) for normalized force, *f_a_*, and *f_xb_* over a range of [Ca^2+^] (Figure 5A–5C) show little difference in Ca^2+^ sensitivity between the two models (Tables 1 and S2; for calculations, see Methods section). As mentioned above, the multi-filament model produces ≈100 times the maximal force with ≈6 times the *f_xb_* as the two-filament model. While there appears to be more variation in force-pCa plots (Figure 5A, where force was normalized to the value at pCa 4 for each model) than in corresponding *f_xb_*-pCa plots (Figure 5C), this is a consequence of normalizing force without changing *f_xb_* calculations from Figure 4. The coefficients of variation between the force-pCa and *f_xb_*-pCa datasets within a given model are nearly identical. Sensitivity to Ca^2+^ (Table 1), is calculated by data fits to a three-parameter Hill equation using pCa as the independent variable (Equation 22). These values for *n_H_* are close to one, as expected in the absence of cooperative feedback between the kinetics of calcium binding to thin filaments and cross-bridge recruitment. The similarities between *n_H_* and pCa_50_ for steady-state force, *f_a_*, and *f_xb_* result from identical thin-filament kinetics between the two models (Figure 3, Table 2). Again this highlights that greater force and *f_xb_* in the multi-filament model result from a difference in geometry between the two models. Thus, sarcomere lattice structure introduces a cooperative mechanism that is independent of Ca^2+^-mediated mechanisms.

**Table 1:**
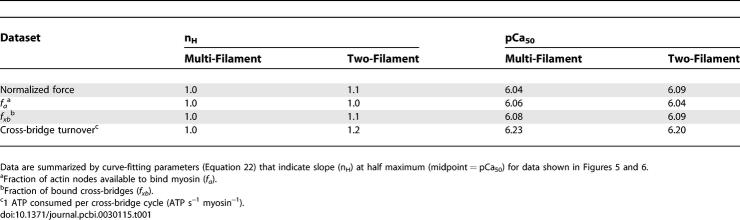
Ca^2+^ Sensitivity for the Multi-Filament and Two-Filament Models

**Table 2:**
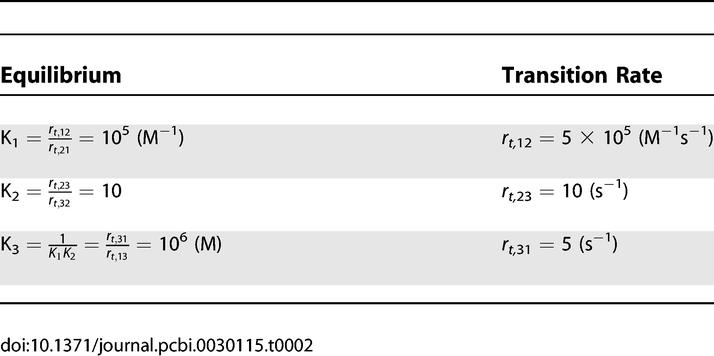
Thin-Filament Transition Rates

**Table 3:**
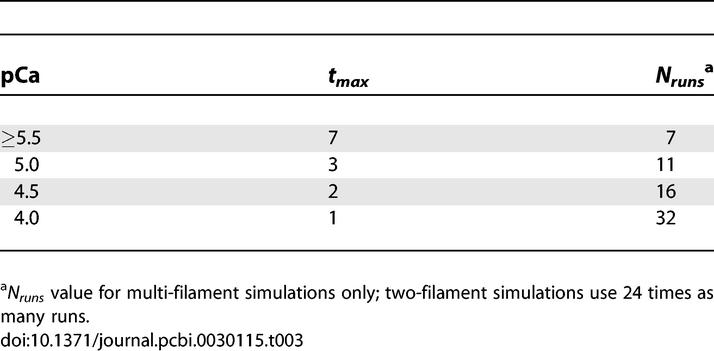
Simulation Parameters across pCa Values

**Figure 6:**
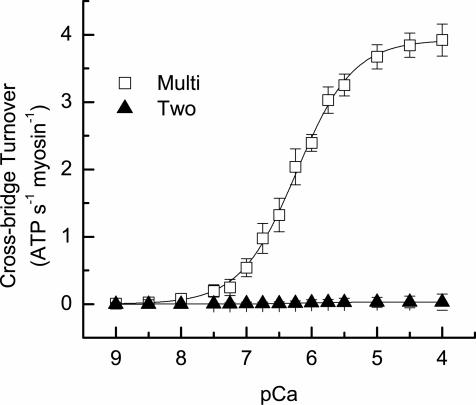
Steady-State Cross-Bridge Turnover Average ATPase (one ATP per cross-bridge cycle) for the multi-filament (open square) and two-filament (black triangle) model is plotted against pCa. Predictions are shown as mean ±SD. When error bars are not visible, they reside within the symbol. Mechanical parameters for *k_xb_*, *k_a_*, and *k_m_* are the same as those listed in the legend of Figure 4. Solid lines represent least squares minimization of ATP consumption to a three-parameter Hill equation (Equation 22).

To determine how model geometry affects the balance of force in each model, we calculated the ratio of steady-state force to number of attached myosin at all simulated [Ca^2+^]. Average steady-state force per bound myosin (Figure 5D) is ≈17 pN for the multi-filament model compared with ≈30 pN for the two-filament model. These predicted values (at *k_xb_* = 5 pN nm^−1^) are greater than experimental estimates of ≈6–8 pN per attached myosin head [35,37], although multi-filament model predictions of force per bound myosin over a range of slightly more compliant *k_xb_* values are more consistent with these experimental estimates (discussed below in detail). These values (Figure 5D) are consistent with estimates for actomyosin rigor bonds [38–40], which may set an upper limit on possible force borne per attached myosin. Two-tailed bootstrap analysis [41] of these results (Figure 5D) indicates a significant slope (*p* < 0.05) for the multi-filament (=−0.56 pN Bound XB^−1^ pCa^−1^) and two-filament models (−8.0 × 10^−9^ pN Bound XB^−1^ pCa^−1^). This slope is much larger for the multi-filament model, indicating an increase in force produced by a bound cross-bridge as [Ca^2+^] increases. This Ca^2+^-sensitive increase implies a coordination between cross-bridge binding and cycling in the multi-filament model that is not observed in the two-filament model.

Steady-state predictions of ATP consumption are greater for the multi-filament model than for the two-filament model for all [Ca^2+^] (Figure 6A). Maximal ATP consumption (pCa 4) in the multi-filament model is 3.9 ± 0.2 ATP s^−1^ myosin^−1^ compared with 0.03 ± 0.12 ATP s^−1^ myosin^−1^ in the two-filament model, ≈135-fold difference. This multi-filament ATPase value agrees well with measured values from skeletal fibers (=3.5 ATP s^−1^ myosin^−1^; [42]). Parameter values for Hill-curve fits on these data show a slightly increased Ca^2+^ sensitivity (pCa_50_, Table 1) for ATP consumption compared with the mechanics predictions in Figure 5A–5C. Normalizing ATPase to the number of myosins directly compares the effect of lattice geometry on cross-bridge turnover rate. This indicates that the ≈6-fold increase in cross-bridge binding (*f_xb_*) in the multi-filament model cooperatively enables ≈135 times the cross-bridge cycling. These results, coupled with the results from Daniel et al. [15], suggest that increased cross-bridge binding and turnover occurs through enhanced compliant realignment of filaments in the lattice. In contrast, low ATP consumption in the two-filament model suggests that cross-bridges are binding, and producing force, but cross-bridge turnover is less frequent. This implies that the two-filament model remains more static, while the multi-filament model exhibits more active realignment between filaments.

Steady-state tension cost, calculated from the quotient of ATP consumption and force, does not significantly differ between the two models at pCa 4 (multi-filament = 0.0041 ± 0.0003 versus two-filament = 0.0057 ± 0.0436 ATP s^−1^ myosin^−1^ pN^−1^). Two-tailed bootstrap analysis [41] of tension cost indicates a small, but significant (*p* < 0.05) slope in the tension cost-pCa relationship (0.001 ATP s^−1^ myosin^−1^ pN^−1^) for the multi-filament model. A similar analysis of tension cost in the two-filament model yields no significant [Ca^2+^] dependence. This result indicates similar mechanisms of individual cross-bridge cycling in each model. Specifically, if an individual cross-bridge binds in either model, it ultimately undergoes a similar range of distortions throughout the cycle.

### Rate of Force Generation

**Figure 7:**
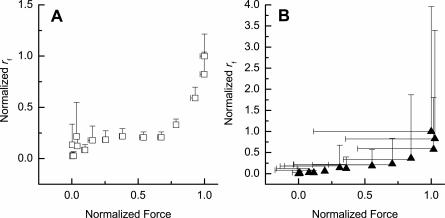
Rate of Force Generation (*r_f_*) Average *r_f_* plotted against normalized force, for the multi-filament (A, open square) and two-filament (B, black triangle) models, is calculated from a single, increasing exponential function ([17], and Methods section). Predictions are shown as mean ±SD, using single-sided error bars along each axis. Error bars that are not visible lie within the symbol. Note the difference in scale on the ordinates.

The two models predict a nonlinear increase in the rate of force generation (*r_f_*) with increasing [Ca^2+^] (calculated as discussed in the Methods section, [17]). Maximal *r_f_* (pCa 4.0) is 48 ± 10 s^−1^ versus 59 ± 176 s^−1^ (mean ±SD) for the multi-filament and two-filament model, respectively. Importantly, the mean values of *r_f_* for a given normalized force are similar for both models. This similarity suggests that individual cross-bridge binding kinetics depend on [Ca^2+^] and force level, but are independent of model geometry. The nonlinear relationship between predicted *r_f_* values versus normalized steady-state force (Figure 7) is similar in shape to measured force redevelopment rates plotted against normalized steady-state force from single, demembranated muscle cell experiments [43–49]. The extraordinarily large SD in the two-filament model predictions follows from an exponential, as opposed to normal, frequency distribution in the set of *r_f_* values.

Though the shape of the *r_f_* -normalized force relationship is similar in both models, the stochastic variation in *r_f_* (error bars in Figure 7) is much less for the multi-filament (A) than the two-filament (B) model. This difference in variation results from greater and more consistent cross-bridge binding at the onset of contraction in the multi-filament model, which follows from a greater number of Ca^2+^-activated actin nodes. For example, within the first few time steps of a simulation at pCa 4 in both models, roughly 50% of the actin nodes are available to bind with myosin (*f_a_*, Figure 4B). This initial *f_a_* level creates a finite duration (≈50 ms) when the thin filament is submaximally activated, leading to spatial inhomogeneities of Ca^2+^-activated regions along the thin filament, even at pCa 4. Building on results discussed above, the likelihood that these few Ca^2+^-activated regions align with a proximal myosin is much greater in the multi-filament versus the two-filament model. Hence, variance in the distribution of initial Ca^2+^-activated actin nodes being spatially available for immediate cross-bridge binding is much less for the multi-filament model. Moreover, any realignment of the filaments following initial cross-bridge binding can increase the probability of additional cross-bridge binding by improving the alignment with these Ca^2+^-activated regions of the thin filament. Thus, increased compliant realignment in the multi-filament model may also help reduce stochastic variation in *r_f_* through increased cross-bridge recruitment.

### Mechanical Tuning of Myofilament Lattice Stiffness Maximizes Force Produced at Saturating [Ca^2+^]

**Figure 8:**
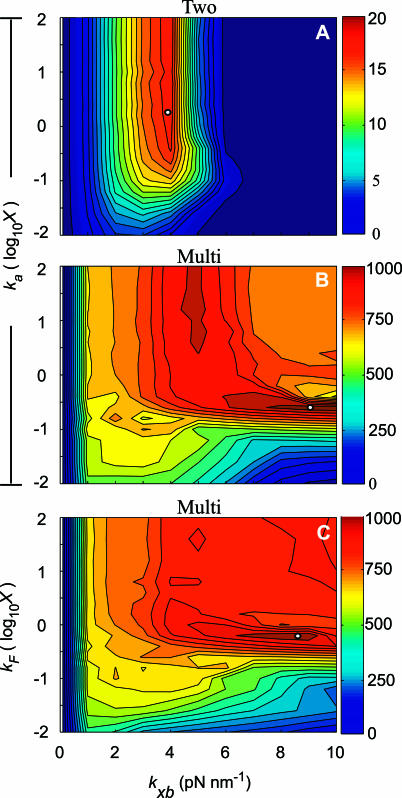
Maximal Force Varies with Lattice Stiffness Contour plots of average steady-state force at maximal Ca^2+^ activation (pCa 4) across a range of mechanical lattice parameters are shown for the two-filament (A) and multi-filament (B,C) models. All simulation predictions adjust *k_xb_* over a range of [0.1–10] pN nm^−1^, shown on the abscissa of each panel. Simulations adjusting *k_a_* and *k_xb_*, while keeping *k_m_* fixed at 6,060 pN nm^−1^ were done for both models (A,B). These simulations adjusted *k_a_* over a four-decade range (with respect to the original value of *k_a_*) using a scalar multiplier, *X*, that ranged from [−2 to 2] in log_10_ space, represented on the ordinate of (A) and (B). (C), however, is a different type of simulation performed only with the multi-filament model. This second type of simulation simultaneously scales the stiffness of both thick and thin filaments (*k_F_*) from their original values, using a similar range of *X* as (A) and (B). Colored-scale bars for force in pN are shown to the right of each panel; note the difference in scale between the two-filament predictions (A) and the multi-filament predictions (B,C). The maximum contour value (white solid circle) is 16, 963, and 933 pN for (A–C).

Myofilament stiffness values influence maximal predicted force (pCa = 4) in both models (Figure 8). To examine the coupling between filament stiffness and predicted force, we used two types of simulations. One set varied thin-filament stiffness (*k_a_*) and cross-bridge stiffness (*k_xb_*), while keeping thick-filament stiffness (*k_m_*) fixed (Figure 8A and 8B). The other set of simulations simultaneously varied both thick- and thin-filament stiffness (*k_F_*) by a scalar factor (*X*), while co-varying *k_xb_* (Figure 8C).

The approach used in the first set of simulations (varying only *k_a_* and *k_xb_*, Figure 8A and 8B) is consistent with prior unregulated models of contraction [15,17], which showed that stiffness of the filament lattice may be “tuned” to maximize predicted force. Generally, varying *k_xb_* in both current models produces little force at the most compliant *k_xb_* values. Force increases to a maximum ridge near moderate *k_xb_* values and then diminishes as *k_xb_* further increases. Note, however, the force dependence on *k_a_* in the multi-filament model (Figure 8B), which forms an L-shaped ridge of maximum force that is not present in the two-filament model (Figure 8A). Additionally, force decreases at higher *k_a_* and *k_xb_* values in the multi-filament predictions (Figure 8B). This decrease differs from the results of Chase et al. [17], where predictions of force continue to rise over increasing values of *k_a_* and *k_xb_*. Our model produces results similar to those of Chase et al. [17] if we increase values of *k_xb_*, but do not correspondingly decrease *xb*_0_ (Equation 13). The contrast between the L-shaped maximum force contour in the multi-filament model and the simple ridge of maximal force in the two-filament model likely follows from increased realignment between compliant filaments in the multi-filament lattice that leads to increased cross-bridge binding at greater *k_xb_* values. This increased range of myofilament stiffness values that produce high force levels in the multi-filament model demonstrates the influence of a cooperative mechanism arising solely from geometry differences between the two current models.

The second set of simulations varied *k_m_*, *k_a_*, and *k_xb_* in the multi-filament model to more fully examine how mechanical properties of the lattice affect force production. This expanded approach (compared with prior studies [15,17] as well as with Figure 8A and 8B) simultaneously varied both thick- and thin-filament stiffness (*k_F_*) by a scalar factor (*X*), while independently varying *k_xb_*. These simulation results show a plateau of high force across a range of stiffer *k_xb_* values that extends from moderate to high *k_F_* values (Figure 8C). This elevated force plateau extends across the stiffest filament and *k_xb_* values (Figure 8C), and thus differs from the L-shaped ridge of elevated force when only *k_a_* varied (Figure 8B). Similar to Figure 8B, the maximal force contour occurs near parameter values that correspond with experimentally derived filament stiffness values [log_10_ *X* = 0] [20–23]. However, there is a slight shift in position of the maximal contour between Figure 8B and 8C. Figure 8C also shows a clearly defined peak of maximal force, in contrast to the ridge of maximal force in simulations tuning *k_a_* independently of *k_m_* (Figure 8B).

### Ca^2+^ Regulation and Myofilament Stiffness Properties Influence Force, Cross-Bridge Binding, and Cross-Bridge Turnover

**Figure 9:**
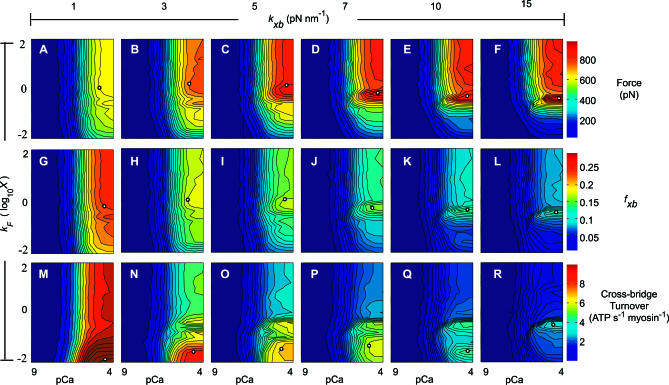
Steady-State Predictions Vary with Lattice Stiffness and [Ca^2+^] Contour plots of average steady-state predictions from the multi-filament model for force (A–F), fractional cross-bridge binding, *f_xb_* (G–L), and cross-bridge turnover (or ATP consumption, [M–R]) as a function of [Ca^2+^], *k_xb_*, and filament stiffness, *k_F_* (simultaneously scaling both thick- and thin-filament stiffness using *X* as in Figure 8C). Within each panel, values of pCa range from 9–4, while *k_F_* values are identical to those in Figure 8C. *k_xb_* increases from left to right across each column of panels as indicated (ranging from 1–15 pN nm^−1^). Contour levels are specified by the color bar at the far right of each row. Areas appearing brown indicate regions where steady-state values exceed the upper limit of the color bar. The solid white circle in each panel corresponds to the maximum contour level, not a single maximum point in pCa-log_10_ *Xk_F_*−*k_xb_* space. The maximum force contour for (A–F) is 683, 766, 897, 949, 885, and 940 pN. Maximal fractional binding is 0.24, 0.18, 0.17, 0.16, 0.14, and 0.13 for (G–L), and maximal ATP consumption is 14.4, 8.57, 6.81, 5.89, 4.58, and 3.74 ATP s^−1^ myosin^−1^ for (M–R).

Multi-filament model predictions of steady-state force as a function of [Ca^2+^], cross-bridge stiffness (*k_xb_*), and filament stiffness (*k_F_*) (uniformly varying both thick- and thin-filament stiffness as in Figure 8C) show that increasing [Ca^2+^] increases force (Figure 9A–9F). Because the model contains no feedback between crossbridge binding and thin-filament activation (*f_a_*) kinetics, *f_a_* is not affected by stiffness properties of the myofilament lattice (unpublished data). For all *k_xb_* values (Figure 9A–9F), there is similar shape to the surface of force produced over the full range of *k_F_* values. Force level is elevated at larger filament stiffness (*k_F_*) values and diminishes with more compliant filament values. Also, greater *k_xb_* values produce a sharper decline in force as filament compliance increases (lower *k_F_* values). The maximal contour value of each plateau (across *k_xb_* values) occurs at *k_F_* values that are similar to experimentally measured values for thick- and thin-filament stiffness [log_10_ *X* = 0] [20–23]. However, the maximal force contour becomes more sharply defined with a peak that shifts slightly toward more compliant *k_F_* values at greater *k_xb_* values (white dots in Figure 9A–9F). The maximal force value for each panel is moderate at compliant *k_xb_* values, increases to a maximum at *k_xb_* pN nm^−1^ (Figure 9D), then slightly diminishes with increasingly stiff *k_xb_*.

Simulations suggest that the number of bound cross-bridges is not directly correlated with the level of force produced. Altering [Ca^2+^], *k_F_*, and *k_xb_* results in the greatest fractional cross-bridge binding (*f_xb_*) at lowest *k_xb_* values (Figure 9G–9L). This maximal *f_xb_* level contrasts with force, which is minimal at lowest *k_xb_* values (Figure 9A–9F). There are general similarities between force production and *f_xb_* as [Ca^2+^] and myofilament stiffness varies: 1) increased [Ca^2+^] increases cross-bridge binding, 2) the maximal contour of each panel occurs at similar *k_F_* values (corresponding to measured values, [20–23]), and 3) the maximal contour width narrows with a shift in the peak (white dots) as *k_xb_* increases. In contrast to force predictions, a plateau of elevated *f_xb_* (over a range of *k_F_* values) exists only at more compliant *k_xb_* values (Figure 9G and 9H), and these plateaus narrow into ridges at greater *k_xb_* values (Figure 9I–9L). Moreover, *f_xb_* consistently declines with increasing *k_xb_* values, although force increases and stabilizes at a high magnitude with increasing *k_xb_* values. Examining the complex correlation between *f_xb_* (Figure 9G–9L) and force (Figure 9A–9F) indicates that relatively small changes in these values can compound to produce larger shifts in estimates of average force borne per bound myosin.

Multi-filament model predictions suggest that cross-bridge turnover decreases with increasing cross-bridge stiffness (one ATP per cross-bridge cycle, Figure 9M–9R). Across all *k_xb_* values, with respect to any specific *k_F_* value, ATPase increases with increasing [Ca^2+^]. In all panels (Figure 9M–9R), the maximal contour of cross-bridge turnover occurs at pCa 4 (white dots), and this maximum shifts toward more compliant filament values with decreased *k_xb_*. Additionally, the peak of the maximum contour becomes sharper as *k_xb_* increases. These results, coupled with force (Figure 9A–9F) and *f_xb_* (Figure 9G–9L), illustrate how force production results from an interaction between mechanical properties of the lattice and kinetics of Ca^2+^-regulated cross-bridge binding.

In summary, comparing results across the panels of Figure 9 show how muscle contraction depends on Ca^2+^-regulated cross-bridge binding within a compliant myofilament lattice. The greatest ATP consumption (Figure 9M) and cross-bridge binding (Figure 9G) occur at *k_xb_* = 1pN nm^−1^, a *k_xb_* value that produces minimal levels of force (Figure 9A). Together, these results suggest that energy consumed at this *k_xb_* value is used to stretch out the filaments, increasing both realignment between compliant filaments and cross-bridge cycling, rather than producing force. On the other extreme, where *k_xb_* = 15 pN nm^−1^, there is a high magnitude of force (Figure 9F), very little ATP consumption (Figure 9R), and minimal cross-bridge binding (Figure 9L), which largely follows from little compliant realignment in the more rigid filament lattice. At intermediate *k_xb_* values, there is a transition between compliant realignment in the filament lattice that coordinates force production versus myosin binding. Comparing all panels in Figure 9 indicates that an optimal lattice stiffness leads to a high ratio of force to ATP consumption (one metric of the energetic consequences of contraction) at *k_xb_* = 3–7 pN nm^−1^, near physiological filament stiffness values (log_10_X = 0).

## Discussion

This study investigated the effect of sarcomere lattice geometry on thin-filament activation, cross-bridge binding, force production, and ATP utilization by comparing two spatially explicit, Ca^2+^-regulated, compliant myofilament models of muscle contraction. The multi-filament model incorporated the physiological ratio of filaments (2–thin:1–thick) in a hexagonal lattice similar to vertebrate striated muscle, while the two-filament model employed a single thin and thick filament. Both models used identical rate functions for thin-filament and cross-bridge kinetics, and neither model incorporated any cooperative feedback between cross-bridge binding and thin-filament activation. The multi-filament model predicts greater force production, cross-bridge recruitment, and cross-bridge turnover with less stochastic variation relative to the two-filament model. These increases are larger than ascribed by the greater number of potential filament interactions in the multi-filament model. Multi-filament model predictions agree better with experimental muscle force and ATPase measurements than two-filament predictions (Table S1), indicating that models including a more physiological representation of sarcomere lattice structure may better analyze the underlying mechanisms responsible for muscle contraction.

Our model geometry produces a filament network representing myosin molecules that directly face thin filaments at hexagonal vertices of the lattice. These geometrical assumptions collapse the three-dimensionality of the system, permitting a linear system of equations to represent filament sliding and force generation in one dimension. This mathematical implementation requires some modifications from known muscle ultrastructure. For example, the thick-filament geometry differs somewhat from vertebrate striated muscle (see the Methods section and Figures S1–S4), and cross-bridge formation in muscle can involve additional degrees of freedom not explicitly addressed by this study (three-dimensional mobility of myosin heads, radial thick- and thin-filament spacing, and rotation or torsion of the thin-filament helices). Other groups are developing three-dimensional models to simulate contraction [50]. However, this study focuses on thick- and thin-filament interactions along the axial direction of the sarcomere lattice and investigates the effect of geometry on two coupled, spatial processes (thin-filament activation and cross-bridge binding) that are important modulators of contraction.

Two central results emerged from this study. First, the ratio and arrangement of thick and thin filaments influences contractile dynamics. Second, contraction depends on an inseparable coupling between geometry, kinetics, and mechanical (stiffness) properties of the myofilament lattice. Interestingly, increases in force production and cross-bridge turnover in the multi-filament versus two-filament model were greater (>100-fold) than the increased number of possible inter-filament interactions resulting from differences in model geometry (24-fold). This indicates a mechanism of cooperative cross-bridge binding that depends on geometry of the myofilament lattice (Figures 4 and 5). The greater force and cross-bridge turnover in the multi-filament model is likely associated with greater realignment within the compliant filament lattice via an increased number of cross-bridge interactions. These findings present a possible consequence of lattice structure in all muscle systems, where variation in the ratio and ultrastructural organization of thick and thin filaments may enhance contractile performance for the given function across different muscle types.

The inseparable coupling between geometry, kinetics, and mechanical (stiffness) properties of the myofilament lattice suggests that spatial, kinetic, or mechanical aspects of muscle function cannot be considered individually when examining muscle performance. This finding follows from simulations co-varying myofilament compliance within two spatial networks while maintaining identical model kinetics (Figures 8 and 9). Importantly, predictions of maximal force occurred at myofilament compliance values near those reported experimentally [20–23]. Together, these two broad findings demonstrate that future studies examining mechanisms of contraction should consider coupling between: spatial behavior of thin-filament regulatory proteins, position and load-dependent cross-bridge cycling, compliant myofilaments, and sarcomere lattice geometry.

### Myofilament Compliance Influences Myosin Force Production and Energy Utilization

Varying the stiffness of cross-bridges and myofilaments alters the relative partitioning of mechanical energy, contributing in part to the behaviors observed in Figure 8. Several molecular phenomena contribute to the tuning observed in these force surfaces as compliance is varied at maximal [Ca^2+^]. The key difference between these simulations is the ridge of high force seen in Figure 8B where thin- (*k_a_*) and thick- (*k_m_*) filament stiffness vary independently. This ridge contrasts with the high force plateau for simulations in which *k_a_* and *k_m_* co-varied (Figure 8C). These simulations suggest that the chemical energy imparted to cross-bridges from ATP hydrolysis is manifest as mechanical energy in the forms of force and deformation within the filament lattice. Thus, for a given amount of energy, some is partitioned as forces transmitted throughout the lattice, and some is partitioned to distortions within the lattice.

To illustrate this point, examine the predicted force with respect to the most flexible link in the filament network (Figure 8). The panels show little force production with very compliant cross-bridges (lower *k_xb_* values), which partitions energy primarily into cross-bridge distortion. Comparably, there is little force produced when either *k_a_* (Figure 8A and 8B) or both *k_a_* and *k_m_* (Figure 8C) are very compliant (for *k_F_*, log_10_ *X* = −2), because energy is partitioned into distortion of compliant filament(s). As cross-bridges' stiffness increases relative to filament stiffness, there is decreased cross-bridge deformation and increased force production. These changes occur through coordinated cross-bridge binding that maintains strain in the filament lattice. Further increasing cross-bridge stiffness forms a ridge of high force as *k_a_* and *k_m_* approach the same order of magnitude (when log_10_ *X* = 0 for *k_a_*) by favorably partitioning energy into both force and lattice distortion (Figure 8A and 8B). This ridge falls off as thin filaments become very stiff in comparison with thick filaments (when log_10_ *X* > 0 for *k_a_* in Figure 8A and 8B), because energy is partitioned primarily into thick-filament distortion. Indeed, this also explains development of the high-force plateau when uniformly scaling both *k_a_* and *k_m_* (Figure 8C), in contrast to the high force ridge when changing *k_a_* alone (Figure 8B).

Two other molecular processes also contribute to the steady-state tuning behaviors; these are recruitment of cross-bridges and their state transitions. As previously reported [15,17], the portion of mechanical energy manifest as lattice distortions alters the position of thin-filament binding sites, thus contributing to an increased probability of cross-bridge attachment. However, high lattice compliance leads to mechanical energy being partitioned almost completely to distortion, and produces little force. Herein lies the crucial tradeoff: distortion allows greater cross-bridge recruitment, but simultaneously decreases the fraction of energy partitioned to force that is distributed throughout the lattice.

**Figure 10:**
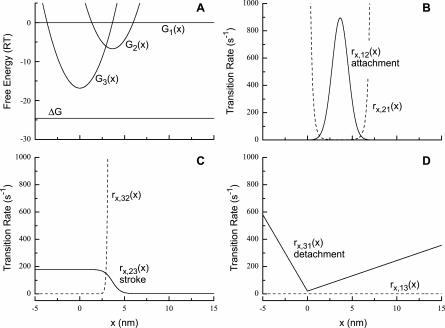
Free Energy and Transition Rate Profiles Position-dependent free energy differences (A) and transition rates (B–D) between cross-bridge states (see Figure 3) are shown for *k_xb_* = 5 pN nm^−1^. The coordinate along the abscissa of each panel, *x*, represents the position difference between a particular pair of actin and myosin nodes associated with cross-bridge formation (Equation 9). (A) Horizontal lines give free energies of detached states (XB1), with the difference between the two horizontal lines representing the standard free energy drop over a full cross-bridge cycle (Δ*G*(*x*), Equation 8). Δ*G*(*x*) is used to define the minimum in each parabolic free energy well *G*_2_(*x*) and *G*_3_(*x*) (representing bound states XB2 and XB3). (B–D) Solid lines are associated with corresponding forward transition rates, and dashed lines are associated with reverse transition rates (Figure 3, Equations 15–17). We define free energies and forward transition rates for each state, then use these to calculate reverse transition rates (Equation 14).

Energy partitioned to lattice deformation controls another crucial feedback mechanism associated with kinetic state transitions. As shown in Figure 10 and in prior studies [3,4,15], the probability of state transitions depends strongly on cross-bridge distortion. Thus, energy imparted to the compliant filament lattice from cross-bridges causes deformation which, in turn, results in cross-bridge distortion. In contrast, cross-bridge binding in an infinitely stiff lattice will have all of the strain-dependent mechanical energy appear as force and none as filament deformation. In this latter situation, all cross-bridges behave independently, with no feedback between cross-bridges to influence additional cross-bridge binding or cycling. Importantly, the mechanism of cross-bridge–induced cross-bridge recruitment requires extensible myofilaments and can only be modeled via spatially explicit methods.

Cross-bridge compliance also contributes to cross-bridge recruitment. Chemical energy from ATP hydrolysis is transformed into mechanical energy in the cross-bridge regardless of stiffness (*k_xb_*). However, *k_xb_* affects the likelihood of a cross-bridge finding an actin site as well as the amount of deformation following binding. This restricts stiffer cross-bridges to bind at nearer sites on the thin filament and may produce higher forces even though distortions will be less. In contrast, a more flexible cross-bridge can bind to more distant regions of the thin filament. These examples illustrate how energy partitioning depends on the stiffness of both filaments and cross-bridges. Thus, smaller deformations associated with stiffer cross-bridges limit additional recruitment of other stiff cross-bridges because they too must be near binding sites, and less compliant realignment of binding sites occurs in a stiffer lattice (Figure 9G–9L).

Lattice compliance also contributes to the ATP utilization associated with cross-bridge cycling (Figure 9M–9R). Simulations that varied myofilament compliance result in high force, moderate cross-bridge binding, and moderate ATP consumption near physiological values of *k_a_* and *k_m_* over a *k_xb_* range of 3–7 pN nm^−1^. Increasing filament or cross-bridge stiffness shows that force remains high with reduced cross-bridge binding and cycling (Figure 9). On the other hand, if the lattice becomes increasingly compliant, minimal force is produced with high ATP consumption. This suggests that an intermediate level of lattice compliance, near physiological values [20–23], optimizes coordinated cross-bridge binding and cycling via compliant realignment of the filament lattice while producing a high level of force with a lower ATP cost.

### Myofilament Lattice Geometry Amplifies Compliant Realignment of Cross-Bridge Binding Sites

Crossbridge–induced crossbridge recruitment results in greater force production and ATPase through realignment of myosin binding sites on compliant thin filaments [15,17]. This effect is amplified by the geometry of the multi-filament (versus the two-filament) model, which more closely reflects the ratio of thick to thin filaments in muscle. Moreover, the augmented force, cross-bridge recruitment, and cross-bridge turnover is larger than would be predicted simply from the greater number of potential interfilament interactions in the multi-filament model. Thus, a cooperative mechanism of contraction arises solely from differences in sarcomere lattice structure.

Even though individual cross-bridges have identical model kinetics, the ensemble average of cross-bridge behavior differs between models (Figures 5–7). The ratio of ATP utilization to force produced is similar between models, which suggests that any single cross-bridge cycle (in either model) preserves the partition of energy from ATP into lattice distortion and force production. Despite this similarity, the force per bound cross-bridge in the multi-filament model is about 40% less than that in the two-filament model (Figure 5D). This likely results from a decrease in the mean distortion of a bound cross-bridge moving through its cycle in the multi-filament model. The decreased mean distortion may result from increased realignment in the multi-filament lattice, which contributes to a decreased force borne by a cross-bridge through the lifetime of a cycle. Alternatively, the increased realignment between filaments in the multi-filament model could enhance coordination between cross-bridges, leading to increased rates of turnover or shifting the temporal distribution of the cycle toward less distorted conformations. Currently, we cannot determine the relative influence from each of these possible mechanisms, as both are intimately coupled given the model kinetics. In any event, the lower force per cross-bridge in the multi-filament model indicates that cross-bridges spend less time in highly distorted configurations and that sarcomere lattice geometry also influences kinetic behavior of cross-bridges.

### Model Geometry Does Not Affect Ca^2+^ Regulation in the Absence of Kinetic Feedback

The component of force generation that is solely a consequence of Ca^2+^ activation of the thin filament is not influenced by sarcomere lattice structure (Figures 4 and 5). A spatially explicit model of regulatory proteins in a system of compliant filaments is an important component of the spatial–temporal coupling between thin-filament activation and cross-bridge binding. Additionally, coupling these two spatial processes is essential to describe the Ca^2+^-dependent amplitude and rate of force development (Figures 4–7). Two important features of the multi-filament model permit investigating contractile dynamics as a function of [Ca^2+^] (compared with previous models [15, 17]): 1) thin-filament kinetics represent Ca^2+^ binding with and dissociating from troponin, interactions between troponin subunits, and movement of tropomyosin, and 2) these activation kinetics are spatially explicit to represent regulatory characteristics of troponin and tropomyosin along the thin filament. These advances introduce a platform to investigate spatial and kinetic molecular mechanisms of cooperativity that may contribute to contraction [11,12,15,27–29].

The current simulations demonstrate a form of cooperative contraction resulting from sarcomere lattice geometry in a system of compliant filaments, but additional forms of cooperativity may result from feedback by cross-bridges or thin-filament regulatory proteins on Ca^2+^ activation or tropomyosin mobility [12,27,29,51,52]. One example of this kinetic feedback may be coordinated movement between adjacent tropomyosin molecules following Ca^2+^ binding with troponin, which activates a region of thin filament greater than the 37 nm length of a single tropomyosin molecule [29]. The similarity of Ca^2+^ sensitivity (pCa_50_) between thin-filament activation, cross-bridge binding, force production, and ATPase in the current models (Table 1) is likely to diverge with cross-bridge and thin-filament–dependent cooperative feedback mechanisms on Ca^2+^ activation, as preliminary work suggests [30–32]. Whether or not any form of kinetic cooperativity is considered in future models of muscle contraction, our results show that the structural determinants of cooperative cross-bridge binding will always play a crucial role in force generation.

## Methods

This study focuses on two Ca^2+^-regulated, spatially explicit models of muscle contraction. Defined above, the multi-filament model consists of four thick filaments and eight thin filaments (Figure 1A). Multiple thick and thin filaments interact in a hexagonal lattice similar to vertebrate striated muscle [53,54]. The two-filament model is a reduced version of the multi-filament model, where myosin molecules and actin monomers only interact along a single plane [15]. Although the simulations discussed in this paper use isometric conditions, half-sarcomere length (=1.2 μm) and filament overlap are controlled variables, similar to previous models [15,17].

### Geometry.

A central assumption restricts interactions between filaments to prescribed regions along thick and thin filaments that directly face each other. This constraint provides a mathematical accounting that enables multiple filaments to interact and reduces a three-dimensional, nonlinear problem into one-dimensional, linear system. These regions of potential interaction represent myosin molecules along thick filaments or myosin binding sites on actin along thin filaments. Dividing these regions into a set of mathematical structures, called nodes, provides a basis of points along the filaments about which forces balance and motions occur.

Two multi-filament model properties permit incorporating hexagonal lattice characteristics of vertebrate skeletal muscle [53,54] (Figure 1A). The first is implementing thick and thin filaments with longitudinal and rotational characteristics to produce co-linear facing rows of actin and myosin nodes that align at hexagonal vertices. As discussed above, this property collapses a higher order problem into a linear problem and allows each thick filament to interact with six different thin filaments while each thin filament interacts with three different thick filaments. The second property is a toroidal boundary condition along the longitudinal axis of the half-sarcomere. Employing this boundary condition at the cross-sectional edges of our simulation wraps each edge onto its opposite edge (Figure 1A). This boundary condition removes any inhomogeneities near the edge of our simulation by eliminating any longitudinal simulation boundary and preserves the 2–thin:1–thick filament ratio within a finite simulation volume. The simple lattice structure depicted in Figure 1A represents myosin filaments coaxially spaced at 40 nm [53]. Thus, the interactions simulated in the multi-filament model represent an 80 (= 2 × 40) nm by 70 (= 2 × 40cos(π/6) ) nm cross-section of infinite lattice space using only four thick filaments and eight thin filaments.

Vertebrate thick-filament structure has three-myosins extending from the filament backbone every 14.3 nm in relaxed muscle (myosin layer lines) [53,55]. Our model preserves this physiological spacing between myosin layer lines along the thick filament, producing a similar number of myosins that can potentially bind actin (=120 multi-filament versus 150 per half-sarcomere length thick filament in vertebrates). Modeled thick filaments (Figures 1 and 2) are 858 nm long and consist of 60 myosin nodes and one node at the M-line to permit position control [17]. Myosin nodes represent myosin layer lines, and the resting, unstrained length between adjacent myosin nodes (*m*_0_) is 14.3 nm. Two myosins extend radially from the filament backbone at each node to form a two-start helix, rotating π / 3 radians every *m*_0_. This thick-filament geometry produces six rows of myosin that project from the center of the thick filament with an overall periodicity of 42.9 nm for the filament. Each row of myosin projects toward a different thin filament. Additional geometric comparisons of different lattice structures are provided in Figures S1–S4.

Each thin filament is 1,119 nm long, containing a total of 90 actin nodes distributed along two entwined actin strands and one node at the Z-line for position control (Figures 1 and 2). Each actin strand has a helical pitch identical to vertebrate striated muscle [= π radians every 37.3 nm, 53]. Actin nodes along each strand are separated by 24.8 nm and rotated by 2π / 3 radians, at rest. The actin nodes represent target binding sites for myosin and provide a spatially explicit accounting for the regulatory proteins to control Ca^2+^-sensitive activation along the thin filament. Similar to physiological thin-filament structure, these two entwined actin strands oppose each other by π radians. We translate the initial node on one strand by 12.4 nm relative to the initial node on the complementary strand, making the nodes rotationally translated by 4π / 3 radians (= π + π / 3; initial offset plus rotation accompanying the 12.4-nm translation). This accounting creates a coiled thin filament where the resting length between adjacent actin nodes (*a*_0_) is 12.4 nm and distributes the 90 actin nodes along three rows (spaced every 37.3 nm along each row). Each row of thin-filament nodes directly faces three different thick filaments.

Controlling thick- and thin-filament interactions via [Ca^2+^] with spatial characteristics of regulatory proteins is a fundamental advancement from previous spatially explicit models [14–18]. The spatial and temporal effects of troponin and tropomyosin are explicitly accounted for in the sections describing model geometry and kinetics. As above, the two actin strands provide a basis for modeling Ca^2+^-activated regions of the thin filament. The spatially explicit model parameter *Tm_span_* represents the influence of tropomyosin by setting the range of adjacent Ca^2+^-influenced regions along each actin strand, effectively determining the number of adjacent actin nodes (i.e., thin-filament length) available for myosin binding. *Tm_span_* represents the effective distance over which Ca^2+^ binding with troponin facilitates tropomyosin movement—activating thin-filament regions where myosin can bind to actin. *Tm_span_* was set at 37 nm in this study, making two adjacent actin nodes along an actin strand available to bind myosin. The first region influenced by *Tm_span_* begins with the first actin node on each strand, making the following region along each strand influence the next two actin nodes on that strand. This accounting scheme continues along the entire thin filament. While preliminary studies [32] varied *Tm_span_* to explore how cooperative mechanisms of thin-filament Ca^2+^ activation may contribute to force generation [29], this study fixes *Tm_span_* to focus solely on the consequences of different sarcomere lattice geometry between models.

### Mechanics.

Mechanics describing simulated force use a system of linear springs (Figure 2) and balance forces at each node in the filament lattice [15,17]. As mentioned above, we model filament sliding and force generation along the longitudinal axis of the half-sarcomere. This assumption collapses the model into a linear system of equations comprising a vector of actin and myosin node positions (*X*), a matrix of spring constants (*K*), and a vector of boundary conditions (*V*). Solving the instantaneous force balance

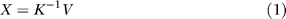
through Gaussian elimination allows us to calculate *X* given known cross-bridge binding conditions throughout the filament network. Individual entries to *K* and *V* result from decomposing Equation 1 into spring constants, rest lengths, and boundary conditions at the filament ends. As with previous models, we also assume that viscous and inertial forces are negligible [15,17,18].

We assign three spring constants, *k_m_*, *k_a_*, and *k_xb_* to the elements between thick-filament nodes, between thin-filament nodes, and between thick and thin filaments following myosin binding to actin (representing the cross-bridge), respectively (Figure 2). Consistent with the earlier two-filament model [15], *k_m_* is ∼1.4 times greater than *k_a_* (= 65 pN nm^−1^ for 1-μm filament length) [20–23]. Most simulations in this study set *k_m_* and *k_a_* at 6,060 and 5,229 pN nm^−1^ to maintain measured filament stiffness values between myosin nodes and actin nodes (for rest lengths *m*_0_ and *a*_0_). Also, most simulations in this study use a *k_xb_* of 5 pN nm^−1^. Although this *k_xb_* value is greater than estimates from single molecule measurements, 0.69–1.3 pN nm^−1^ [25,56], it is closer to estimates of 3–5 pN nm^−1^ from muscle fiber measurements [34–37]. Using *k_xb_* = 1pN nm^−1^ in previous models and in this study resulted in relatively low predicted force (compared with *k_xb_* = 5 pN nm^−1^ [15, 17], Figures 8 and 9), suggesting that a parameter value of 5 pN nm^−1^ better estimates *k_xb_* than 1 pN nm^−1^. We recognize that *k_xb_* is a fundamental myosin property contributing to the chemomechanical energy transduction and force produced in muscle. Therefore, running a large number of simulations characterized the effect of *k_xb_* on predicted force, fractional myosin binding (*f_xb_*), and cross-bridge turnover (Figure 9). This approach illustrates how *k_xb_* influences simulations across the range of estimated values (1–5 pN) listed above, while recognizing the variability and difficulty associated with exactly specifying this parameter value. The resting distortion of a myosin cross-bridge (*xb*_0_) is directly linked to *k_xb_* (Equation 13), and no stiffness parameters (*k_m_*, *k_a_*, or *k_xb_*) depend on [Ca^2+^] as suggested by Isambert et al. [19].

The instantaneous sum of forces at each actin or myosin node in the network is zero, independent of any actomyosin binding. As described above, the system of linear equations describing this force balance uses spring constant and position information between all connected nodes. Force development and any corresponding realignment in the filament network may distort the distance between nodes from specified rest lengths. Generally, each term in the equations below (Equations 2–4) contributes to the force balance as a Hookean spring element of stiffness *k_m_*, *k_a_*, or *k_xb_* with a distortion from corresponding rest length *m*_0_, *a*_0_, or *xb*_0_. As further described below, *xb*_0_ (Equation 13) represents the unbound rest length where myosin S1 heads (assuming coincident behavior of the two S1 heads per modeled myosin molecule) position is offset from its corresponding myosin node. The balance of forces about myosin node at position *m_j_* oriented to bind with co-linear facing actin node at position *a_i_* (depicted in Figure 2):

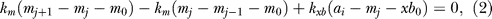
where *m_j_*_−1_ and *m_j_*_+1_ represent the position of myosin nodes adjacent to *m_j_* along the thick filament. Equation 2 is written for a coordinate system defining positive force to the right, such that *m_j_*_−1_ < *m_j_* < *m_j_*_+1_. The first and second terms of Equation 2 balance forces along the thick filament, while the third term accounts for the interaction between thick and thin filaments associated with cross-bridge formation. If there is no cross-bridge binding, the third term disappears from Equation 2 (*k_xb_* = 0). A similar balance of forces occurs about the actin nodes at position *a_i_* and *a_k_* (Figure 2, where *a_i_*_−1_, *a_i_*_+1_, *a_k_*_−1_, and *a_k_*_+1_ are positions of actin nodes adjacent to *a_i_* and *a_k_* along each respective thin filament:

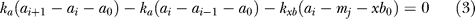


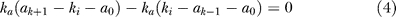



Following any cross-bridge binding in the network, forces balance throughout the lattice causing myofilaments to deform or realign. Any local distortion and node realignment in the system affects the balance of force throughout the entire network.

Force per filament is calculated using distortion (difference from rest length) in the spring element nearest the Z- or M-line (Δ*x_i_*). Total force at the Z- or M-line (*F_Z-line_* or *F_M-line_*, respectively) is calculated by summing over the number of filaments in parallel at the ends of our half-sarcomere simulation:

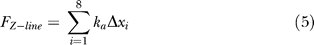
and

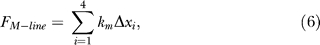
where *F_Z-line_* = − *F_M-line_*.

### Kinetics.

Model kinetics (Figure 3) use a three-state cross-bridge cycle coupled with a three-state, [Ca^2+^]-sensitive, thin-filament regulatory cycle (Figure 3). Cross-bridge kinetics are distortion-dependent, as with previous models [1,3,4,10,15,17,57]. The kinetics of each cross-bridge depends upon the behavior of all other cross-bridges through coupled interactions within the compliant filament lattice [15]. While more complete chemomechanical descriptions of cross-bridge cycling would require an increased number of biochemical states [3,4,6,10], we continue using a three-state cross-bridge model to directly compare with earlier modeling efforts [15,17].

Previous models suggest that a minimum of three mechanical states is required to characterize actomyosin binding and force production [15,17]: an unbound or weakly bound, nonforce-bearing state (XB1), a state where myosin binds to actin in a conformation preceding the mechanical transition, often referred to as the powerstroke (XB2), and a state where myosin is bound to actin in a conformation following the powerstroke (XB3) (Figure 3). The pre-powerstroke state (XB2) should contribute less force to the myofilament lattice than the post-powerstroke state (XB3), similar to the two-attached states outlined by Eisenberg et al. [3]. However, the actual force borne by any cross-bridge (= *k_xb_*(*a_i_*−*m_j_*−*xb*_0_) (in Equation 2 or Equation 3, see Figure 2) depends on its distortion from rest length.

Concomitant with each mechanical state is a biochemical state representing the cyclical hydrolysis of ATP, release of inorganic phosphate (P*_i_*) and ADP, and binding of another ATP that leads to dissociation of myosin from actin Figure 3. These states represent a collapsed version of larger biochemical schemes [6]. The nonforce-bearing state (XB1) corresponds to a biochemical state where myosin binds the ATP hydrolysis products ADP and P*_i_* and is unbound or weakly bound to actin. In the pre-powerstroke state (XB2), the actomyosin complex is formed with myosin having ADP and P*_i_* bound. While it remains debated whether P*_i_* is released before, concurrent with, or following the powerstroke, the post-powerstroke state (XB3) represents an actomyosin conformation where myosin has released P*_i_* and only ADP is bound [6,58–62]. The transition back to the nonforce-bearing state entails myosin releasing ADP, binding ATP, and dissociating from actin.

Cross-bridge elasticity [1] imposes position-dependent transition rates throughout the cross-bridge cycle. Elastic sliding between filaments creates either a positive or negative force exerted by the cross-bridge and depends upon cross-bridge distortion [3]. Consistently, the current state transitions intimately couple distortion of a myosin molecule with filament realignment throughout the lattice (Figure 10). While the thermodynamic and kinetic parameters describing cross-bridge cycling in the model are exactly the same for each myosin, the geometry and mechanical coupling between myosins and filaments does not allow myosin molecules to function independently [15]. The specific functions defining cross-bridge free energies and transition rates are presented below.

Biochemical and structural studies demonstrate that both spatial and temporal thin-filament processes regulate actomyosin binding [7]. Thin-filament regulation results from interactions between Ca^2+^ binding to troponin, and subsequent movement of tropomyosin exposing myosin binding sites on actin to allow cross-bridge cycling [63]. A structural regulatory unit spans 37.3 nm along each actin strand of thin filaments, containing one troponin and tropomyosin that covers seven actin monomers. Thus, Ca^2+^ binding to each troponin will expose only a local region of myosin binding sites along each actin strand in proximity to the troponin complex. The coupled mechanical and structural properties of myofilaments further influence actomyosin binding along the filaments, where up to two myosin can potentially bind per 37.3 nm actin strand. Hence, the spatial and kinetic processes of thin-filament activation and cross-bridge cycling are inseparable.

Two key events underlying the thin-filament regulatory model are Ca^2+^ binding to troponin and the ensuing interaction between troponin and tropomyosin (Figure 3). We simulate these events along each actin strand of the thin filament in conjunction with *Tm_spam_* (introduced above in the geometry section). This method directly links spatial and kinetic characteristics of troponin and tropomyosin to regulate actomyosin binding, which is unique to this model. While portions of the thin filament may be activated, whether any binding occurs depends on myosin proximity (Equation 15).

The thin-filament kinetic model (Figure 3) employs three states [51,63], with transition rates defined below (Table 2). In the first state (TF1), no Ca^2+^ is bound to troponin and actin nodes are unavailable to bind cross-bridges. The second state (TF2) has Ca^2+^ bound to troponin, and actin nodes remain unavailable to bind cross-bridges. In the third state (TF3), Ca^2+^ is bound to troponin and actin nodes are available to bind with myosin. The equilibria-associated thin-filament state transitions (*K*_1_, *K*_2_, or *K*_3_) adhere to:

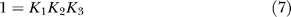
maintaining thermodynamic stability [64]. Each equilibrium equals the ratio of forward (*r_t_*_,*ij*_) to reverse (*r_t_*_,*ji*_) thin-filament transition rates.

### Two-filament model.

The two-filament model in this study is a subset of filament interactions from the multi-filament model. The simulations employ the same thick- and thin-filament geometry of the multi-filament model, but permit interactions between only one thick and one thin filament. Mathematically, this reduces the multi-filament model interaction to a single row of myosin and actin nodes that co-linearly face each other [15]. This results in fewer inter-filament interactions: 20 myosin nodes and 30 actin nodes. In all other regards (geometry, kinetics, and mechanics), the two-filament model is identical to the multi-filament model.

### Implementation algorithm.

All simulations were programmed using Matlab (version 7.0, The Mathworks, http://www.mathworks.com). Monte Carlo simulations use a fixed time step (Δ*t*) of 1 ms, and state transitions were accepted by comparing *p_ij_*(= *r_ij_*Δ*t*) to a random number generated from a uniform distribution [15,17]. Simulations evaluate kinetics at all actin and myosin nodes and calculate the resulting force balance at each time step. At each time step, the algorithm scans through each region of thin-filament activation (set by *Tm_span_*) using thin-filament kinetics and Monte Carlo methods to determine the thin-filament state associated with each actin node. Following the thin-filament query, the algorithm scans through myosin nodes using Monte Carlo methods to determine cross-bridge state transitions based on cross-bridge kinetics, proximal actin node availability, and filament position. Finally, the program calculates the effects of these kinetic transitions on myofilament realignment to determine the position of all actin and myosin nodes to begin the next time step according to Equation 1.

### Kinetic rates.

The total free energy liberated over a complete actomyosin cycle (Δ*G*) depends upon the standard free energy of ATP hydrolysis (Δ*G*_0,*ATP*_) and the concentration of ATP, ADP, and *P_i_* [3,4,34]:

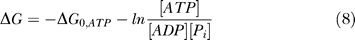



Similar to previous models [3,4,15,17], Equation 8 defines all free energies in units of RT, where R is the ideal gas constant and T is temperature in Kelvin. Δ*G*_0,*ATP*_ = 13RT at 300K [4], and we set [ATP] = 5 mM, [ADP] = 30 μM, [Pi] = 3 mM, and T = 288K, which makes Δ*G* ≈ 24 *RT*. Free energies for the nonforce bearing (*G*_1_), pre-powerstroke (*G*_2_ ), and post-powerstroke (*G*_3_ ) states depend on distance (*x*) and cross-bridge stiffness (*k_xb_*_,*RT*_), in units of RT nm^−2^ (which converts to pN nm^−1^ using an appropriate scale factor). *x* is calculated from the position difference between a myosin node and its nearest available actin node. Using the example depicted in Figure 2 for myosin extending from myosin node at position *m_j_* and binding to actin node at position *a_i_*:

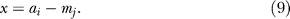



Following binding *x* determines cross-bridge distortion and consequently its contribution to the force balance (Equation 2, *k_xb_*(*a_i_* − mj − *xb*_0_) = *k_xb_*(*x* – *xb*_0_)). Free energy functions define elastic behavior in each cross-bridge state (Figure 10A):

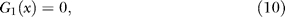


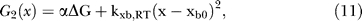


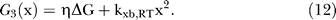



*G*_1_(*x*) arbitrarily sets the reference energy at 0 RT to begin an ATP hydrolysis cycle. Because no force is borne by this state, the energy profile is independent of cross-bridge distortion. The parabolic functions *G*_2_(*x*) and *G*_3_(*x*) depend on *k_xb_*_,*RT*_, representing elastic behavior of a myosin extending from a node positioned at x = 0 in Figure 10A. The free energy difference between *G*_1_(*x*) and the minimum of each well represents the maximal amount of energy that myosin can convert to work in either force-bearing state. These energies are proportional to Δ*G*, set by *α* = 0.28 and *η* = 0.68, representing free energy drops between M-D-P*_i_* and A-M-D-P*_i_* consistent with previous models [3,4,15].

Cross-bridge distortion following hydrolysis of ATP,

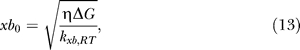
is constrained by myosin stiffness and the free-energy of ATP hydrolysis, illustrated by the position offset between the minimum of *G*_2_(*x*) and *G*_3_(*x*). The energy profile associated with the pre-powerstroke conformation results from thermal fluctuations about *xb*_0_, where mechanical energy is stored in the myosin molecule. Ultimately, this energy stored in the cross-bridge is transferred into the filament lattice to produce force and lattice realignment or filament sliding. The degree of realignment (as well as the correlated “powerstroke” distance) becomes a function of local cross-bridge and filament strain, balanced throughout the network.

Forward (*r_x_*_,*ij*_) and backward (*r_x_*_,*ji*_) transition rates between cross-bridge states *i* and *j* (Figure 3) employ free-energy estimates defined above (Figure 10A) to maintain a detailed thermodynamic balance [4,15,17]:

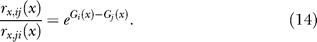



We use Monte Carlo methods to calculate probabilities of state transition (*p_ij_*): *p_ij_ = r_ij_* Δ*t*, where Δ*t* is the time step of the simulation, and *r_ij_* is the rate of the transition in question [15,17]. The actomyosin binding rate (*r_x_*_,12_) follows from Daniel et al. [15]:

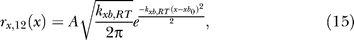
(Figure 10B), where *A* is assigned the numerical value of 2,000. The dimensions of *A* and all subsequent scale constants below (*B*, *C*, *D*, *M*, *N*, *P*) are set to yield transition rates in s^−1^. Because most simulations herein set *k_xb_* = 5 pN nm^−1^ (five times more than previous simulations [15,17]), which may have underestimated *k_xb_*), we doubled *A* to produce similar likelihoods of myosin binding as these prior studies.

Recent measurements suggest that filament strain determines cross-bridge transition rates [24,26]. The effects of elastic strain energy (*E_strain_* = *k_xb_x* ^2^) on cross-bridge rate functions were originally considered by Daniel et al. [15] for *r_x_*_,12_ only. Here, we reformulate the rates representing the powerstroke transition (*r_x_*_,23_) and cross-bridge detachment (*r_x_*_,31_) to include cross-bridge distortion and stiffness dependencies:

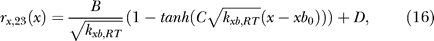
where *B*, *C*, and *D* take numerical values of 100, 1, and 1, respectively. Reformulating *r_x_*_,23_ permits greater transition probability with decreasing cross-bridge stiffness (Figure 10C).

Similar to previous models [1,4,15], cross-bridge detachment rate is distortion-dependent, with an increased rate for negative distortions. To ensure that the distortion-dependent detachment rate is invariant with cross-bridge strain energy for any *k_xb_* value, *r_x_*_,31_ scales proportionally with 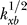 :

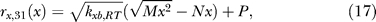
where *M*, *N*, and *P* take numerical values 3600, 40, and 20, respectively, and

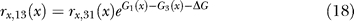
(Figure 10D). Any reverse transition associated with *r_x_*_,13_ is unlikely because it requires energy input. The scale constants listed above were selected to preserve the kinetic behaviors outlined in previous models [15,17].

Rates of both Ca^2+^ binding to troponin and subsequent protein interactions leading to tropomyosin movement are not fully defined in striated muscle, but some information is available from solution measurements. Additionally, some estimates of these rates are based on force development and relaxation kinetics in muscle. Thin-filament transition rates for this study are listed in Table 2. The transition rate representing Ca^2+^ binding to troponin (*r_t_*_,12_) becomes a second-order rate transition, dependent upon [Ca^2+^]. This second-order property activates the thin filament more slowly with lower [Ca^2+^], reducing the rate of thin-filament activation from pCa 4.0 to 7.5 in Figure 4. The value of *r_t_*_,12_ lies in the range of literature values derived from solution biochemistry [65–68] and unpublished data from the Regnier lab, but may be slower in the presence of the organized filament lattice. Two equilibria represent quick Ca^2+^ binding and slower thin-filament activation: *K*_1_ represents a fast-equilibrium Ca^2+^ binding, and *K*_2_ is a slower equilibrium representing troponin–tropomyosin interactions [65,69,70]. Ca^2+^ dissociation rate (*r_t_*_,31_) is taken from muscle-relaxation studies and adjusted to represent rates of force relaxation with respect to force generation [71]. Preliminary simulations investigating cooperative force production [30] used only a two-state thin-filament regulatory cycle (Ca^2+^ on/off). Under this two-state regulatory scheme, it was difficult to control Ca^2+^ sensitivity in the force–pCa relationship, and we concluded that three states better represent the thin-filament regulatory system. This agrees with experiments suggesting that differences in skeletal and cardiac regulatory proteins are partially responsible for differences in cooperative force production between the two muscle types [28,72,73].

### Simulation parameters.

Setting the maximum simulation time (*t_max_*) for each [Ca^2+^] ensured that force, cross-bridge binding, and thin-filament activation reach their equilibrium values. Although the spatially explicit nature of our model does not permit an analytic solution, *t_max_* is derived from the time-independent solution of a simplified linear differential equation model for thin-filament activation (see Figure 3):

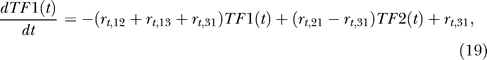


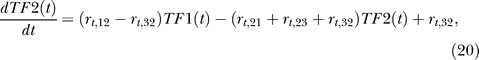


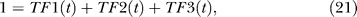
where *TF*1(*t*), *TF*2(*t*), *TF*3(*t*) represent the fraction of actin nodes in each state.

Averaging the final 10% of each simulation run (a single [Ca^2+^] with time range 0 to *t_max_*) extracts the steady-state, asymptotic value for that run. Previous experience with acceptable standard deviations of steady-state values [15,17] helps maximize computational efficiency by calculating the number of runs (*N_runs_*) required at each [Ca^2+^]. *N_runs_* ensures that the set of steady-state averages at each [Ca^2+^] (gathered from the last 10% of each run) is generated using no fewer than 3,200 total time steps (Table 3).

Simulation parameters and data reduction methods used for the two-filament simulations are identical to the multi-filament model, with the exception of *N_runs_*. Preliminary tests indicated that two-filament simulations using exactly the same *N_runs_* as the multi-filament model do not provide a reasonable level of certainty for simulation predictions. Increasing *N_runs_* in the two-filament model by a factor of 24, chosen to represent the increased number of inter-filament interactions between the two models, improves model output through increased numerical averaging and provides a scale factor consistent with geometry differences.

### Data reduction.

During each run, simulations record force and ATP consumption, as well as position and kinetic state for actin and myosin nodes. The reported steady-state means and corresponding standard deviations are calculated from the set of *N_runs_* asymptotic averages at each [Ca^2+^] (Table 3). Similarly, the reported rates of force generation (*r_f_*, Figure 7) and their corresponding standard deviations are calculated at each [Ca^2+^] from the set of exponential fits for each run [17]. Averaging the means from each run and calculating the standard deviation at each [Ca^2+^] focuses on the variation between runs resulting from stochastic kinetics rather than on variations within runs. Data points within a run are not independent because cross-bridge transition probabilities determined in successive time steps depend on the prior history of cross-bridge behavior. Each run is independent of other runs. Calculations of force per cross-bridge (Figure 5D) and tension cost subsample the set of asymptotic averages by discarding runs without any cross-bridge binding at the low [Ca^2+^]. If there is no cross-bridge binding, there is no force production or ATP consumption, so the resulting calculation is singular.

Steady-state data are fit to a three-parameter Hill equation:

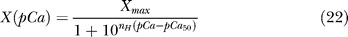
using a nonlinear least squares minimization of *X*(*pCa*). Depending on the dataset in question, *X*(*pCa*) represents: normalized force (Figure 5A), fraction available (*f_a_*, Figure 5B), fraction bound (*f_xb_*, Figure 5C), or ATP consumption (Figure 6). *X_max_* represents the maximal value of the Hill relationship for the dataset in question. pCa_50_ represents the midpoint of the saturation curve and is a measure of Ca^2+^ sensitivity in the system. *n_H_* is the slope of the Hill curve at pCa_50_ and represents the cooperativity in the system. For all fits, we minimize residuals using the entire set of asymptotic averages at all pCa levels across all runs.

## Supporting Information

Figure S1 Hexagonal Lattice Overview of the Multi-Filament Model A cross-section of the modeled multi-filament lattice, similar to Figure 1. Large, red circles represent thick filaments, and small, blue circles represent thin filaments. The thick, black square approximates the modeled region. Thin black lines connecting thick and thin filaments represent co-linear facing rows of myosin or actin nodes, indicating vertices where inter-filament interactions may occur. This structure provides an overview for the accounting method that prescribes which co-linear facing nodes can interact in the model.
(468 KB PDF)

Figure S2 Hexagonal Lattice in the Multi-Filament Model (A–C) Show details of the two-start, thick-filament periodicity, where any node (A) is followed by adjacent thick-filament nodes 14.3 nm (B) and 28.6 nm (C) along the filament. To maintain thick-filament periodicity every 43.3 nm (as in vertebrate striated muscle), adjacent thick-filament nodes rotate π / 3 radians every 14.3 nm. Figure components representing the thick and thin filaments, myosins, and the approximate region modeled are as described in the legend of Figure S1.
(482 KB PDF)

Figure S3 Hexagonal Lattice in the Simple-Lattice Model (A–C) Show the detailed three-start, simple-lattice, thick-filament geometry using a similar configuration as described by Figure S2. However, three-start helical properties leave only 1/3 of the cross-bridges co-linearly facing thin filaments [54]. Any myosin node (A) is followed by adjacent nodes 14.3 nm (B) and 28.6 nm (C) along the filament. There is a 2 π / 9 radian rotation between each adjacent myosin node, maintaining thick-filament periodicity every 43.3 nm.
(485 KB PDF)

Figure S4 Hexagonal Lattice in the Super-Lattice Model (A–C) Show the detailed three-start, super-lattice, thick-filament geometry has a slightly altered configuration compared with Figure S3. While any single thick filament is identical to the three-start properties shown in Figure S3, every other thick filament is rotated by π / 3 radians with respect to the neighboring filaments to accommodate super-lattice properties [54].
(486 KB PDF)

Table S1 Comparisons of Predictions to Published Experimental Values (36 KB DOC)

Table S2 Parameter Values from Hill Fits to Steady-State Model Predictions (28 KB DOC)

Text S1 Alternative Multi-Filament Model Geometries (28 KB DOC)

## Abbreviations

*f*_*a*_: fraction of modeled actin sites activated by Ca^2+^ binding to the thin filament
*f*_*xb*_: fraction of modeled myosin (cross-bridges) bound to actin
*k**_a_*: spring constant for modeled thin-filament elements
*k*_*m*_: spring constant for modeled thick-filament backbone elements
*k*_*xb*_: spring constant for modeled myosin
*xb*_0_: resting (unbound) distortion of myosin
*x*: distortion of the myosin
