# Supplementary material for: Sarcomere Lattice Geometry Influences Cooperative Myosin Binding in Muscle
Source: PLoS Comput Biol. 2007 Jul 13;3(7):e115. doi: 10.1371/journal.pcbi.0030115 (PMC1914368; doi:10.1371/journal.pcbi.0030115)
Supplement: Supplementary file 1 [file pcbi.0030115.sg001.pdf]

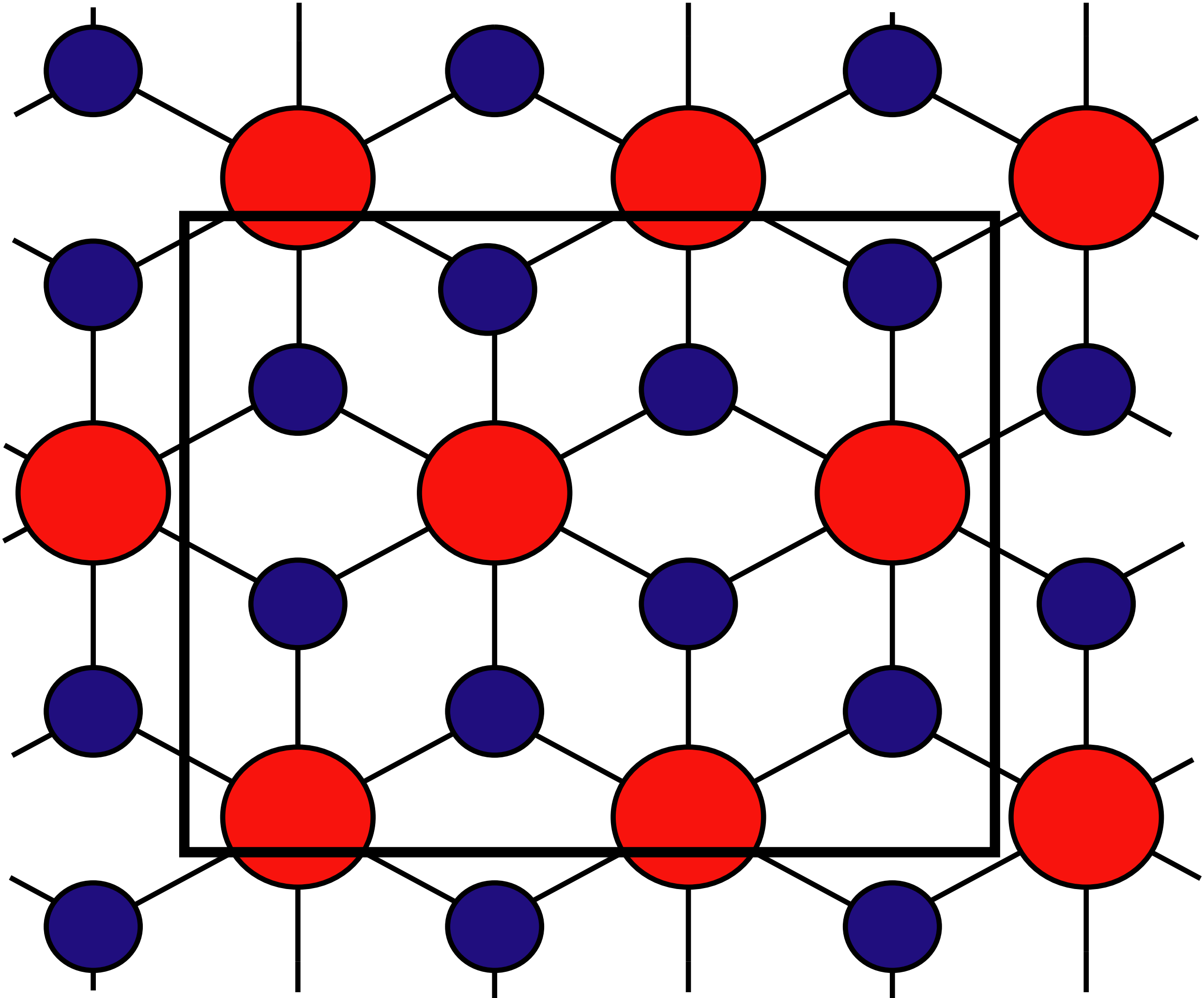

Figure S1
