# Supplementary material for: Sarcomere Lattice Geometry Influences Cooperative Myosin Binding in Muscle
Source: PLoS Comput Biol. 2007 Jul 13;3(7):e115. doi: 10.1371/journal.pcbi.0030115 (PMC1914368; doi:10.1371/journal.pcbi.0030115)
Supplement: Supplementary file 5 [file pcbi.0030115.sd001.doc]

## Supporting Text: Alternative multi-filament model geometries

Initial model comparisons used a series of multi-filament models (listed below) with identical kinetics describing thin filament activation and cross-bridge cycling. Each model also maintained a hexagonal lattice packing for the thick and thin filaments, similar to vertebrate striated muscle. Thin filament geometry remained exactly as described in the associated manuscript. The difference between these models was the helical structure and lattice orientation of the thick filaments:

1.	The **Multi-filament model** was the focus of the associated manuscript. This model used a 2-start helix to construct the thick filaments. Further detail of this geometry is discussed in the supplemental text (Figs. S1 and S2).

2.	The **Simple-lattice model** used a 3-start helix to construct thick filaments (Fig. S3). These thick filaments were oriented in a simple-lattice structure within the hexagonal filament lattice [54].

3.	The **Super-lattice model** used a 3-start helix to construct thick filaments (Fig. S4). These thick filaments were oriented in a super-lattice structure within the hexagonal filament lattice [54].

Vertebrate thick filament structure has three-myosins extending from the filament backbone, forming a three-start helix that rotates 2π/3 radians every 42.9 nm in relaxed muscle [53, 55]. This physiological structure yields ≈150 myosins per half-sarcomere length thick filament, of which only 1/3 directly face a thin filament. Three-dimensional protein flexibility provides that all of these myosins can potentially bind to actin. While these interactions occur within a hexagonal filament lattice, vertebrates have two types of lattice structure: simple-lattice and super-lattice [54].

All published spatially-explicit models of muscle contraction examine one-dimensional behavior along the filaments using a system of linear equations [14-18]. Similar to these models, we only consider filament interactions that occur between co-linearly oriented actin and myosin nodes. This constraint precluded binding by 2/3 of the modeled myosins in either of the three-start models (simple-lattice and super-lattice).

The two-start, multi-filament model used a geometry where all 120 myosins per thick filament have some probability of binding actin, resulting in better model approximations of force, cross-bridge binding, and cross-bridge turnover than the simple-lattice or super-lattice models (Table S1). While multi-filament model geometry was described in the associated manuscript, Figs. S1 and S2 further outline the detailed thick filament geometry in the multi-filament model. Simple-lattice geometry (Fig. S3) used a regular hexagonal packing of thick and thin filaments [54]. Super-lattice geometry (Fig. S4) applied a π/3 radian rotation to half of the thick filaments, making every thin filament approached by at least one thick filament [54]. With the exception of this rotational orientation between thick filaments in the super-lattice model, all helical properties were conserved within thick filaments between the simple-lattice and super-lattice models. All simulation methods for the simple-lattice and super-lattice models were identical to the multi-filament model methods described in the associated manuscript.

The primary differences between these models were the steady-state, average values for force, fractional myosin binding (f), and ATPase (Table S1). The low f and ATPase values in the simple-lattice and super-lattice models indicate less coordination between cross-bridge binding and cycling, compared to the multi-filament model. Again, there was no significant differences in Ca sensitivity between all four models (Table S2), as discussed for the multi-filament versus two-filament models in the associated manuscript. Given the linear constraints of these models, we suggest that the multi-filament model is better suited than the other models to investigate potential mechanisms of cooperativity in muscle.
