# Supplementary material for: Sarcomere Lattice Geometry Influences Cooperative Myosin Binding in Muscle
Source: PLoS Comput Biol. 2007 Jul 13;3(7):e115. doi: 10.1371/journal.pcbi.0030115 (PMC1914368; doi:10.1371/journal.pcbi.0030115)
Supplement: Supplementary file 6 [file pcbi.0030115.st001.doc]

| **Supplemental Table 1**- Comparisons of predictions to published experimental values. | | | | | |
| --- | --- | --- | --- | --- | --- |
|  | ATPase^#^ | *f_xb_^*^* | Model force^‡^ | Relative force^**^ | Stress^†^ |
| Multi | 3.9 $\pm$ 0.2 | 0.101 $\pm$ 0.002 | 958.5 $\pm$ 32.3 | 2 $\pm$ 0.07 | 171 $\pm$ 6 |
| Two | 0.03 $\pm$ 0.12 | 0.015 $\pm$ 0.014 | 9.2 $\pm$ 8.2 | 0.46 $\pm$ 0.41 | - |
| Simple | 1.4 $\pm$ 0.1 | 0.033$\pm$.001 | 436.1 $\pm$ 23.4 | 0.61 $\pm$ 0.03 | 77 $\pm$ 4 |
| Super | 1.6 $\pm$ 0.1 | 0.034$\pm$.002 | 450.5 $\pm$ 23.7 | 0.63 $\pm$ 0.03 | 80 $\pm$ 4 |
| Published | 3.5[42] | 0.1-0.2[34,36] | - | 1.7-2.8[25,34] | 175-256[29,36,42] |
| ^#^ 1 ATP consumed per cross-bridge cycle (ATPs^-1^ myosin^-1^) | | | | | |
| ^*^ fraction of bound cross bridges (*f_xb_*) | | | | | |
| ^‡^ total model force (pN) | | | | | |
| ^**^force per total myosin (pN myosin^-1^) | | | | | |
| ^†^Tensile stress (force per cross-sectional area). All multi-filament models represent an area of 5600 nm^2^ (1kPa=1mN mm^-2^). The area represented by the two-filament model is undefined. | | | | | |
