# Supplementary material for: Sarcomere Lattice Geometry Influences Cooperative Myosin Binding in Muscle
Source: PLoS Comput Biol. 2007 Jul 13;3(7):e115. doi: 10.1371/journal.pcbi.0030115 (PMC1914368; doi:10.1371/journal.pcbi.0030115)
Supplement: Supplementary file 7 [file pcbi.0030115.st002.doc]

**Supplemental Table 2-** Parameter values for Hill fits to steady-state model predictions^*^.

| Data Set | n_H_ | | | | pCa_50_ | | | |
| --- | --- | --- | --- | --- | --- | --- | --- | --- |
|  | Multi | Two | Simple | Super | Multi | Two | Simple | Super |
| Normalized force | 1.03 | 1.08 | 0.95 | 1.06 | 6.04 | 6.09 | 6.06 | 6.11 |
| *f_a_^†^* | 1.01 | 1.01 | 1.01 | 1.0 | 6.06 | 6.04 | 6.04 | 6.05 |
| *f_xb_^‡^* | 1.02 | 1.08 | 0.95 | 1.04 | 6.08 | 6.09 | 6.08 | 6.12 |
| Cross-bridge turnover^**^ | 1.0 | 1.2 | 0.98 | 1.01 | 6.23 | 6.2 | 6.16 | 6.15 |
| ^*^Data are summarized by curve fitting parameters (Eq. 22) that indicate slope (*n_H_*) at half maximum (midpoint=*pCa_50_*) for data shown in Fig. 5 and Fig.6*.* | | | | | | | | |
| ^†^fraction of actin nodes available to bind myosin (*f_a_*) | | | | | | | | |
| ^‡^fraction of bound cross-bridges (*f_xb_*) | | | | | | | | |
| ^**^1 ATP consumed per cross-bridge cycle (ATP s^-1^ myosin^-1^) | | | | | | | | |
